# Benefits and barriers in a clinical research competency development scheme for low- and middle-income countries

**DOI:** 10.1080/16549716.2022.2035504

**Published:** 2022-03-24

**Authors:** Núria Casamitjana, Mahnaz Vahedi, Sarah Davoren, Eleni Kavoura, Joan Tallada, Sara Yamaka, Pascal Launois

**Affiliations:** a ISGlobal, Hospital Clínic - Universitat de Barcelona, Barcelona, Spain; b Special Programme for Research and Training in Tropical Diseases (TDR), Geneva, Switzerland

**Keywords:** Training, mentorship, clinical research, capacity strengthening, evaluation

## Abstract

**Background:**

The EDCTP-TDR Clinical Research and Development Fellowship (CRDF) scheme has offered one-year clinical research training placements for early- and mid-career researchers from LMIC since 1999.

**Objective:**

Using the results of a 2018 external evaluation of the CRDF, the current article aims to identify the principal benefits for the main stakeholders of the CRDF scheme as well as the main barriers to accessing these benefits.

**Method:**

Data analysis was derived from an external evaluation of the CRDF scheme. Based on a logical framework approach, data for the external evaluation was collected through document review, interviews, focus groups, and questionnaires collected from the main stakeholder groups. The evaluation was structured along six main themes: relevance, effectiveness, efficiency, impact, sustainability, and equity.

**Results:**

The current paper focuses on the expected benefits, unexpected benefits, and barriers to enjoying benefits of the scheme for key stakeholders.

**Discussion:**

Expected benefits were aligned with the development of clinical research competencies, which is the objective of the scheme. Unexpected benefits centred on transferable professional skills in scientific leadership and knowledge translation. Barriers mainly were found around engagement with home institutions and the return and reintegration of fellows following the training period.

**Conclusions and Recommendations:**

Recommendations include further engagement with and support for home institutions and developing a formal framework for the development of transferable professional competencies, including leadership and knowledge transfer competencies.

## Background

To achieve scientific equity in health research, persistent gaps in individual and institutional capacity and infrastructure between high-income countries (HIC) and low- and middle-income countries (LMIC) must be addressed. These gaps are prejudicial to research, including on infectious diseases, which often affect the most vulnerable and hard to reach people within LMIC. Clinical research is considered vital for improving health outcomes in LMIC but is still frequently conducted and led by HIC-based institutions. Although shifting the concentration of clinical research from HIC has been a focus of international partnerships for more than 20 years, major barriers, including those related to research capacity, remain in place in many countries, and there are many challenges to developing clinical research in LMIC settings that have not been adequately addressed [1]. LMIC leadership in clinical research is fundamental to generate and implement appropriate solutions for the health needs of their populations and, for this reason, it is essential that international organisations and partnerships prioritise developing scientific capacities [2].

### Development of the clinical research and development fellowships scheme

The Special Programme for Research and Training in Tropical Diseases (TDR), located at the World Health Organisation (WHO), was created in 1974 to strengthen research capacity in LMIC. In 1999, TDR launched the then called Career Development Fellowship through a partnership with GlaxoSmithKline (GSK) Biologicals (Belgium), which remains one of the scheme’s principal Training Partner Organisations (TPOs). Between 1999 and 2007, nine fellows were selected, all of whom were assigned to a one-year training placement at GSK. During 2008–2017, the Bill & Melinda Gates Foundation (BMGF) became the principal funder of the scheme and supported an increase in the number of fellows and in the number and type of TPOs, to include, in addition to pharmaceutical companies, product development partnerships (PDPs) and research institutions. In 2014, the European & Developing Countries Clinical Trials Partnership (EDCTP) joined as a partner in what is now called the EDCTP-TDR Clinical Research and Development Fellowships (CRDF) [3–5].

The CRDF scheme is open to early- to mid-career candidates employed by a legal entity in an LMIC in which they conduct clinical research activities relevant to TDR’s scope. Fellows are selected through a competitive call and assigned to a one-year supervised and mentored training period with a TPO to develop skills relevant to clinical research and product development for malaria, tuberculosis, and neglected tropical diseases, including scientific management, regulatory compliance, and good health research practices. At the conclusion of the training period, fellows return to their home institutions.

**Figure 1.**
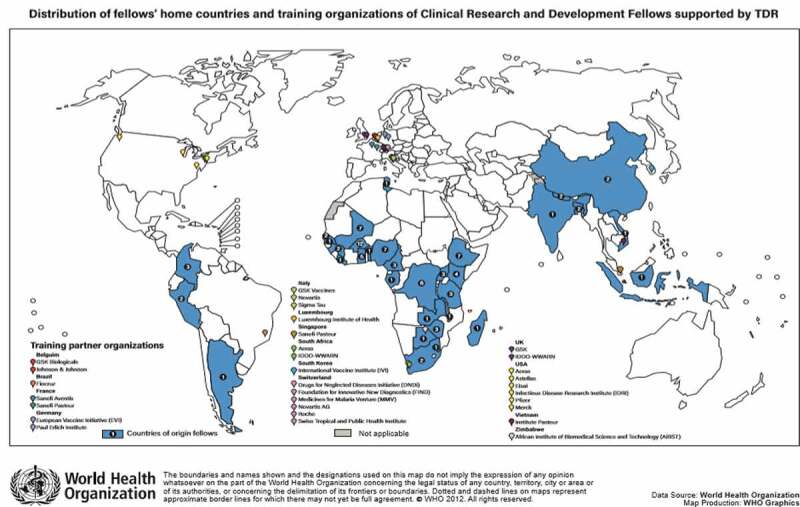
Map of CRDF home countries and training partner organisations, 1999–2017.

Between 1999 and 2017, 91 fellows were placed for training and completed the CRDF scheme (Figure 1). The fellows represented 33 countries with 82% from the WHO African Region.

### External evaluation of the CRDF scheme

In 2012, an external evaluation of the scheme was completed, and the results were published as a research article [6]. In 2018, a new external evaluation of the CRDF scheme was carried out by the Barcelona Institute for Global Health (ISGlobal – Barcelona, Spain), with the objectives of the development and use of a performance assessment tool for improving the CRDF scheme, including its gender equity and its partnerships with TPOs and other partners; and an appropriate and relevant impact evaluation at regional level in order to measure the critical mass of scientists with enhanced research capabilities, who after returning to their home institutions will apply and share the skills learnt.

Using the results of this external evaluation, the current article seeks to:

1. Identify if the main CRDF stakeholders are receiving the expected benefits from their participation, based on the objectives of the scheme;
2. Identify unexpected benefits to participants in the CRDF scheme; And
3. Describe potential barriers to stakeholders translating the benefits gained from the scheme to their scientific environments.

## Methods

The data analysed here were generated from the external evaluation of the CRDF scheme completed in 2018, which was structured using a logical framework approach. The model was based on that used for the previous 2012 evaluation of the scheme [6], for consistency and in agreement with TDR. The six main themes and questions were:

1. **Relevance**: Is the CRDF scheme addressing important challenges, needs, and gaps in LMICs?
2. **Effectiveness**: How effective is the current management of the programme in providing the expected training and capacity development?
3. **Efficiency**: Is the programme cost-efficient to deliver the proposed training and capacity development scheme?
4. **Impact**: How has the CRDF scheme contributed to increasing the clinical research and development capacity of scientists and institutions in LMICs?
5. **Sustainability**: Is the programme sustainable over time, both in terms of financing and partnership?
6. **Equity**: Is the programme equitable in terms of gender, geographical, and language distribution, and institutional participation?

The first four of the six themes and key questions identified in the framework (Relevance, Effectiveness, Efficiency, Impact) were used in the 2012 evaluation and also included in the 2018 evaluation for purposes of comparability. The last two (Sustainability and Equity), were agreed upon with the CRDF scheme management, based on their programmatic priorities.

A lack of well-structured indicators and evidence for the impact of capacity strengthening activities in LMIC has been documented, as have been strategies and frameworks intended to facilitate improved evidence generation and analysis [7,8]. In this context, the external evaluation was oriented around the observations of three key stakeholder groups: CRDF fellows, home institutions, and training partner organisations (TPOs), which participated in the scheme during the evaluated period. This was particularly the case in terms of the Relevance, Effectiveness, Impact, and Equity themes and less so in the case of the Efficiency and Sustainability themes, which focused on programme management and budget allocation.

In addition to using TDR internal documents, such as databases, pre- and post-training plans, re-entry proposals and reports, Scientific Working Group reports, and publicly available documents, such as reports, articles, and information available via the TDR Fellows’ website located on The Global Health Development Network, evaluators surveyed CRDF scheme stakeholders for whom they had a working email address, using an online questionnaire via Google Forms. Of a total of 91 current and former fellows, 89 were contacted. Of a total of 71 home institutions, 57 were contacted. Of a total of 29 TPOs, 27 were contacted.

The same questionnaire was tailored to survey each of six stakeholder groups:

1. All TPOs
2. Home institutions without a current fellow
3. Home institutions with a current fellow
4. Former fellows who participated in the 2012 evaluation
5. Former fellows who did not participate in the 2012 evaluation
6. Current fellows

Fellows, home institution supervisors, and TPO supervisors were asked via email for their consent to participate in the evaluation. Those who consented were then sent an email containing a link to the questionnaire, which was completed online via Google Forms. Fellows were incentivised to participate in the survey with a prize drawing sponsored by WHO/TDR/Research Capacity Strengthening in which two fellows would be awarded 3,000 USD to be used toward attendance at an international conference.

The response rate for fellows was 65%. For home institutions, the response rate was 25% and for TPOs, 48% (Table 1). The particularly low response rate from home institutions may introduce bias into the survey results from this group.

**Table 1.** Response from surveyed groups

	**Total**	**Contacted** (email address available)	**Responded**	**Response rate** (responded/total)
**Fellows**	**91**	**89**	**59**	**65%**
**Home Institutions**	**71**	**57**	**18**	**25%**
**TPOs**	**29**	**27**	**16 (14)**	**48%**

There were 16 responses to the TPO questionnaire from supervisors, representing 14 institutions. This is because in two cases, two supervisors from the same institution responded to the survey. All 16 responses are considered in data analysis.

Subsequently, evaluators conducted interviews and focus group discussions (Table 2) with key stakeholders, both face-to-face and via teleconference. Interviews and focus group discussions took place after the collection and initial analysis of the questionnaires. In selecting countries for site visits the evaluators considered: (1) the number of fellows and number of home institutions in each country, (2) the availability of different types of stakeholders to participate in interviews, and (3) security risks implied in organising travel. The evaluators attempted to create a balanced representation from the perspective of gender, as well as country and region when inviting fellows to interview. However, practical issues were also considered, including optimising the costs of travel by organising trips to countries with higher numbers of former CRDF fellows, and managing security risks, which may have created inherent bias in data collected from the interviewees.

**Table 2.** Interviews and focus groups

Type	Subject	No.
Focus group discussion	Former and current fellows	2
Interview	Former fellows	12
Interview	Home institution	6
Interview	TPO	6
Interview	TDR	1
Interview	Global Health Network	1
Interview	EDCTP	1

External evaluators visited three sub-Saharan African countries to interview fellows and home institutions in addition to Geneva, Switzerland, to interview fellows, a TPO representative, and scheme organisers. Interviews of stakeholders located in other countries took place via teleconference. In at least two cases, the home institution supervisor interviewed was not the fellow’s original supervisor at the time of the training period, but rather the current supervisor at the time of the interview. Interviewed TPO supervisors were selected based on the first analysis of questionnaires, seeking a balance of TPO types represented (pharmaceuticals, PDPs, or research institutions).

Interviews and focus group discussions with fellows, TPOs, home institutions, and other institutional representatives were conducted using a semi-structured approach with a predefined interview guide and template (Annex A). The interviews were audio recorded and then transcribed according to the template. Responses were coded so they could be anonymised, analysed, and used without identifying the individuals interviewed.

The current article focuses on an analysis of data gathered in the context of the external evaluation of the CRDF scheme, undertaken in 2018. It focuses on the results from the Relevance, Effectiveness, Impact, and Equity themes as presented in the evaluation report, which are the themes most closely aligned with the experiences of fellows, home institutions, and TPOs. The current article does not analyse results derived from the Efficiency and Sustainability themes developed in the external evaluation, which were more closely aligned with the interests and goals of the CRDF scheme’s management and funders.

## Results

Expected benefits, unexpected benefits, and the potential barriers to stakeholders enjoying benefits from participation in the CRDF scheme were identified by analysing the results of the aforementioned 2018 external evaluation, with a particular focus on the perspective of the scheme’s stakeholders, especially fellows and home institutions.

Expected benefits at the individual level are mainly constituted by improvements in the clinical research competencies that are at the core of the CRDF scheme’s objective of increasing clinical research competency in LMIC. Here, the expectation is that CRDF fellows would acquire a higher level of competency on a range of clinical research skills areas, which may differ slightly from fellow to fellow based upon their professional and academic backgrounds and the nature of their CRDF training placement.

The benefits expected for home institutions are mainly constituted by improvements to clinical research outcomes based on the re-incorporation of the trained fellow to institutional research activities, as well as on the returned fellow’s contributions to institutional capacity through research capacity strengthening activities with their colleagues.

Unexpected benefits are defined here as benefits to individuals and institutions that are not directly related to clinical research competencies. Barriers are the presence or absence of institutional structures or supports that allow CRDF fellows to fully develop the skills and competencies acquired, and for home institutions to incorporate and maximise the positive effects of their participation in the scheme.

### Expected benefits

#### Stakeholder perceptions of CRDF scheme relevance

As a precursor to addressing potential benefits from CRDF participation, the relevance of the scheme’s objectives to LMIC home institutions and TPOs (in their role as research collaborators and partners) is pertinent to understanding if needs and priorities for advancing LMIC clinical research are reflected in the scheme itself.

Through the survey, home institution representatives were asked to identify key institutional challenges in their research and working environments from a predefined list, as well as the degree to which the CRDF scheme addresses those challenges. TPO representatives were asked to identify key challenges that they face when working with research institutions in LMIC, and the degree to which the CRDF scheme addresses those challenges.

Fifty percent or more of responding home institution representatives (n = 10) identified *funding* (100%), *lack of collaboration with other institutions/companies* (70%), *staff knowledge/capacity* (60%), and *not enough staff with experience in institution* (60%) as challenges. Fifty percent or more of responding TPO representatives (n = 16) identified *facilities/infrastructure* (75%), *staff knowledge/capacity* (69%), *regulatory issues* (63%), and *funding* (50%) as challenges.

On average, home institution and TPO representatives rated the degree to which the CRDF addresses *staff knowledge/capacity* at 3.8 and 4, respectively, on a scale from 1 (not at all) to 5 (fully). Home institution representatives, on average, rated the degree to which the CRDF addresses *lack of collaboration with other institutions/companies* at 3.6, and TPO representatives rated the degree to which it addresses *regulatory issues* at 3.1. The degree to which the CRDF scheme addresses other key challenges, including *funding* and *infrastructure* (issues not directly addressed by the scheme), was, on average, rated below 3 by both stakeholder groups.

In this sense, although the CRDF scheme was not perceived to effectively address some of the highest priority institutional challenges, such as funding and facilities/infrastructure, it does address to a high degree (3.8–4 out of 5) staff knowledge and capacity, the challenge with which the scheme’s objective is most closely aligned.

#### Stakeholder perceptions of CRDF scheme effectiveness

The evaluators asked fellows and home institutions to report their observations of the impact of participation in the CRDF scheme on individual development of clinical research competencies as well as on the translation of those competencies to the institutional environment. In the context of the CRDF scheme, professional competencies for clinical research were defined by the TDR Global Competency Framework for Clinical Research [9,10].

Eighty-two per cent of CRDF fellows who responded (n = 44) reported a self-assessed improvement on specific skills and competencies after participation in the CRDF scheme (of either ‘better’ (42%) or ‘much better’ (40%)) (Table 3).

**Table 3.** Fellows’ self-assessed change in competency level in clinical research skills and competencies from survey questionnaires

**Competencies**	**No Change** Number of responses (percentage of responses)	**Better** Number of responses (percentage of responses)	**Much Better** Number of responses (percentage of responses)	**Better + Much Better** Number of responses (percentage of responses)	**Total responses**
Protocol operationalization (developing study plans, QMS, SOPs, CRF and DMS)	0 (0%)	22 (50%)	22 (50%)	44 (100%)	44
Design & planning of research (health related research developing methodology, protocol, attracting funding)	1 (2%)	23 (52%)	20 (45%)	43 (98%)	44
Interpretation of study results (analysing data, dissemination of results in publications, etc.)	1 (2%)	26 (59%)	17 (39%)	43 (98%)	44
Quality assurance (GCP, QMS controlling quality of the research)	4 (9%)	17 (39%)	23 (52%)	40 (91%)	44
Professional skills (managing a team, organisational skills, record keeping, IT skills, work ethic)	4 (9%)	23 (52%)	17 (39%)	40 (91%)	44
Safeguards (Ethics, safety and risk management, insurance and liability needs)	6 (14%)	10 (23%)	28 (64%)	38 (86%)	44
Study communications (reporting to a funder, facilitating meetings, liaison skills)	6 (14%)	19 (43%)	19 (43%)	38 (86%)	44
Regulations and governance (securing or maintaining approvals and contracts, regulatory approvals)	7 (16%)	17 (39%)	20 (45%)	37 (84%)	44
Oversight (starting and closing a study, project management, monitoring)	8 (18%)	14 (32%)	22 (50%)	36 (82%)	44
Data flow (creating and managing a database, collecting accurate data)	12 (27%)	20 (45%)	12 (27%)	32 (73%)	44
Resources management (financial management, logistics and facilities management, documenting work)	13 (30%)	20 (45%)	11 (25%)	31 (70%)	44
Staff management (Human resources, designing and delivering training, supervising or mentoring)	14 (32%)	16 (36%)	14 (32%)	30 (68%)	44
Interaction with public & study participants (engaging with the community, enrolling and retaining participants, informed consent)	14 (32%)	16 (36%)	14 (32%)	30 (68%)	44
Clinical and laboratory operations (providing clinical care, IMP use, handling biomedical products, performing laboratory assays)	20 (45%)	14 (32%)	10 (23%)	24 (55%)	44
**Total number of responses (percentage of responses)**	**110 (18%)**	**257 (42%)**	**249 (40%)**	**506 (82%)**	**616 (100%)**

According to the policy of the CRDF scheme, at the start of the placement, all fellows complete a training plan together with their TPO supervisor to establish the clinical research competencies that will be addressed during the training period, as well as the fellow’s self-assessed level of competency (again, referencing the TDR Global Competency Framework for Clinical Research) on a scale of 0 to 5, as defined by

1. **No experience**
2. **Trained** (Have received training but have no personal experience in this task or activity)
3. **Some experience** (Have performed this task or activity but not regularly or recently (less than one year’s experience or occasional or past experience))
4. **Capable** (Capable in this task or activity, it is part of my job and I am competent with approximately 1–2 years’ experience)
5. **Experienced** (Consistently competent at this task or activity. It is a normal part of my job and I can conduct it confidently with no supervision)
6. **Highly experienced** (Have been performing this task or activity for many years, and play a leading role in it)

At the conclusion of the training period, fellows re-assess their level of competency. For practical purposes, training plans are often modified during the training period. The evaluators analysed a subset of 12 training plans from fellows who had submitted complete plans, both pre- and post-training. At the start of the training period, the 12 fellows assessed their competencies at an average of 1.54, a score that falls between *trained* (1) and *some experience* (2), according to the aforementioned scale. At the end of the training period, the 12 fellows included in this analysis assessed their competency at an average of 2.83, a score that is 0.17 points below *capable* (3), according to the scale. The average increase of 1.28 is aligned with expectations for a one-year training period, according to the TDR Global Competency Framework for Clinical Research (Table 4) [9].

**Table 4.** Fellows’ self-assessed level of competency, by competency category, from pre- and post-fellowship training plans

Competency categories	Average score at start (A) (n = 12)	Average score at end (B) (n = 12)	Increase (B-A)
Clinical trial reporting	1.45	2.83	1.38
Clinical trials operations and study implementation	1.68	2.85	1.17
Communication and teamwork	1.50	2.68	1.18
Data management and informatics	1.94	3.17	1.23
Ethical considerations and patient safety	2.02	3.12	1.09
Leadership and professionalism	1.37	2.37	1.00
Medicines development and regulation	1.29	2.68	1.39
Safety evaluation and risk management	0.50	2.88	2.38
Scientific concepts and research design	1.58	2.73	1.14
Study and site management	2.11	3.00	0.89
**Average overall scores for all competency categories**	**1.54**	**2.83**	**1.28**

**Figure 2.**
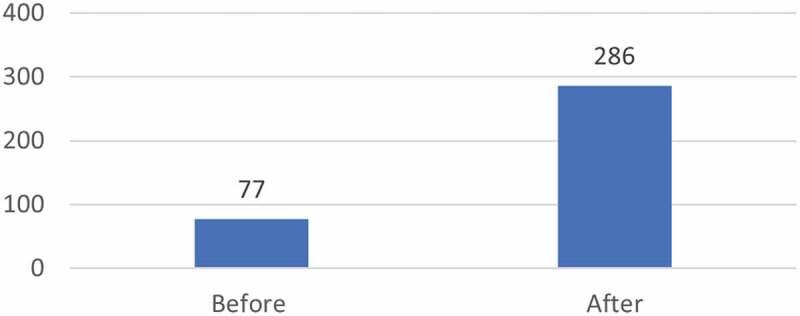
TDR analysis of number of CRDF fellows’ publications before and after placement (2002–2014).

**Figure 3.**
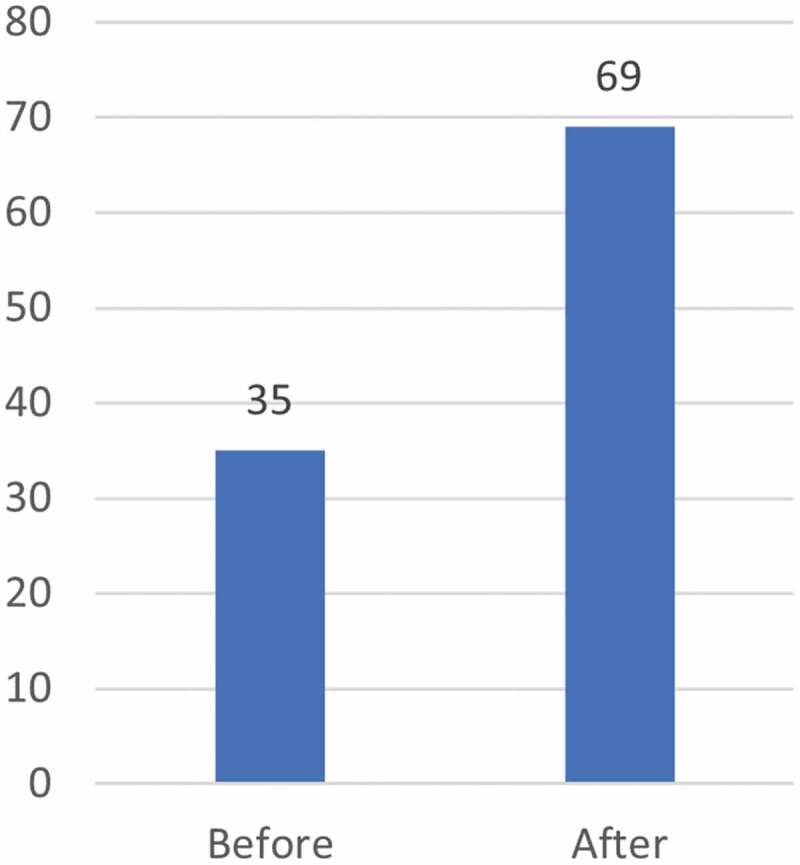
TDR analysis of number of CRDF fellows’ first-authored publications before and after placement (2002–2017).

The evaluators additionally had access to a TDR analysis of CRDF fellows’ publications in peer-reviewed journals (2002–2014). While this analysis shows an increase in both overall number of publications and in first authorship (Figs 2, 3), the degree to which these increases can be attributed to fellows’ participation in the scheme is limited as multiple other factors may contribute to the detected increase.

#### Benefits for home institutions

One of the key factors for impact from the perspective of home institutions is whether fellows return to work, bringing with them, applying, and sharing their newly acquired or improved competencies. While return to the home institution is a requirement of the CRDF scheme, this does not mean that returned fellows will stay for a long period or that their return will necessarily have the intended impact.

The evaluation found that most CRDF fellows returned to their home country and/or region. Three fellows (5%) who responded to the survey (n = 59) reported that they had left their home country/region following the training period. In two of these cases, the fellows had entered a PhD programme (located in a HIC) following their participation in the scheme. In contrast, almost 23% of fellows (10/44) reported having left their home institution. Of those who left, seven stayed in their home country, and two remained in the same region, although they left the country.

All home institution representatives (n = 10) reported that returned fellows had provided training to their colleagues (Table 5).

**Table 5.** Number of training activities carried out by returned fellows as reported by home institutions from survey

Number of trainings delivered at home institution by fellow after return	Percentage of home institutions (n = 10)
2	40% (4)
3	20% (2)
4	20% (2)
or more	20% (2)

**Figure 4.**
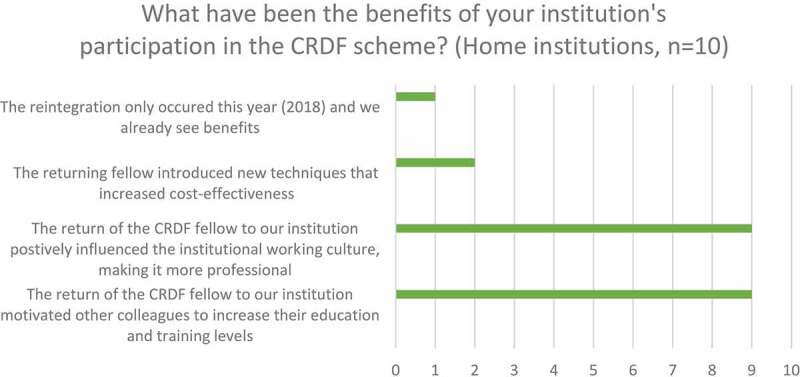
Home institution perception of institutional benefits of participation in the CRDF scheme from survey.

Ninety per cent of home institutions (n = 10) affirmed that the return of CRDF fellows motivated other colleagues to increase their education and training level and positively influenced the institutional working culture (Figure 4).

Other improvements perceived by home institution representatives included an increase in the number of grant proposals prepared and sent and increased attendance at conferences and congresses (Table 6). However, fewer home institution representatives reported that participation in the CRDF scheme led to an increase in the number of clinical trials in which they participate (40%) or number of clinical trials where their institution participates as a coordination site (10%) (Table 6). In any case, it is difficult to attribute increased participation in clinical trials to the CRDF scheme alone, given that other factors, like available funding for clinical trials, site, and country population characteristics, etc., may contribute here.

**Table 6.** Self-reported improvements at home institutions following participation in the CRDF scheme from survey

Improvements at home institutions	% of home institutions that reported perceiving improvement (n = 10)
Led to an increase in the number of proposals for funding prepared and sent to funders.	80% (8)
Led to an increase in attendance to conferences and congresses by members of institution.	70% (7)
Led to the number of scientific peer reviewed publications, where institution is affiliated to an author, increased because of participation in the CRDF scheme.	60% (6)
Led to an increase in the number of research projects that have been granted funding.	50% (5)
Led to an increase in the number of clinical trials in which they participate.	40% (4)
Led to an increase in the number of clinical trials where your institution participates as a coordination site.	10% (1)

### Unexpected benefits

While the survey focused on the development of clinical research skills and competencies, in interviews fellows emphasized the professional skills that form the core of the TDR Global Competency Framework for Clinical Research [9], improved confidence in scientific performance and leadership skills, and an increase in the level of respect they received from colleagues as important outcomes of their CRDF experience. Some fellows related perceived gains in scientific leadership and knowledge translation, including writing and publishing scientific articles, writing grant proposals, communication, leading scientific meetings, networking, and developing training activities, which often occurred in the context of their relationship with a TPO mentor or supervisor.

These competencies, while not the main focus of the scheme, are partially included in the TDR Clinical Research Competencies as ‘Professional Skills’ and were highly valued by all stakeholders and seen as impactful in terms of professional development [9].

### Barriers

Barriers to enjoying the benefits of the CRDF scheme become evident on the return of fellows to their home institutions. Following the 2012 evaluation, in an effort to mitigate problems reported by fellows on return to their home institutions [6], the CRDF launched a re-entry grant scheme aimed at supporting the capacity strengthening activities of returned fellows. In the current evaluation, surveyed home institution representatives (n = 10) observed that the grants had been useful for *reintegration* (average 4.6/5), *human resources strengthening* (average 4.3/5), and the *development of research networks* (average 4.4/5), as well as for giving visibility to the returned fellow and placing value on their experience. Accordingly, surveyed fellows (n = 31) who reported having received a re-entry grant (74%) responded that grants were useful to support *reintegration* (average 4.5/5), *human resources strengthening* (average 4.2/5), and *developing networks with other groups* (average 4.3/5).

Although re-entry grants are only intended for use in supporting capacity strengthening activities, fellows and home institutions expressed interest in re-entry grants to support research instead of training. In some cases, fellows had to relinquish a re-entry grant when they changed jobs or entered a PhD programme.

About 50% of fellows reported that they had moved into a better position in their home institution after the fellowship and many reported seeing their responsibilities increased, although these career advancements can be influenced by multiple factors.

Although there were improvements seen from the 2012 evaluation, about 21% of fellows still reported facing integration obstacles when returning to their home institutions.

As identified in the first evaluation, a lack of ownership and engagement in the CRDF scheme on the part of home institutions continues to be a possible barrier to both individual and institutional benefits from participation. This lack of connection may be evidenced, for example, by the low response rate of home institutions to the external evaluation.

Equity in the scheme is also worth addressing as a potential barrier to benefits at both individual and institutional levels, particularly in terms of how fellows and home institutions are selected to participate in the scheme. The current evaluation analysed equity issues in the CRDF scheme along the lines of gender (individual level), country, and institutional development. Particular attention was drawn to the urgent necessity of addressing the significant gender imbalance in fellows. Sixty-nine men and 22 women (24%) participated in the scheme as fellows during the evaluated period, somewhat below the estimated global average of women in research [11], although this percentage may reflect the reality of gender balance within participating regions, countries, or institutions. Strengthening women’s clinical research capacities and scientific leadership is a stated priority for TDR and, in the context of global discussions on women leaders in global health, TDR has since cosponsored a crowdsourcing challenge funded by BMGF with UNICEF, UNDP, The World Bank, and WHO, to identify creative and feasible ideas to increase the number of women in TDR mid-career clinical research fellowships [12]. As a result of the implementation of suggestions, identified in the challenge contest during the third selection of fellows in 2020–2021, 41.5% of the identified candidates for interviews with TPOs and 50% of finally selected participants were women.

In terms of geographical and institutional equity, some stakeholders expressed the view that CRDF should adopt a strategic approach to offering fellowship placements to researchers from peripheral, or less well-positioned research institutions, rather than rewarding those working at already well-resourced entities.

## Discussion

Based on the results of the 2018 external evaluation of the CRDF scheme, it can be affirmed that the scheme’s objectives are relevant to its key stakeholders (fellows, home institutions and TPOs) as they are aligned with key research institutional challenges. In this sense, staff knowledge/capacity is addressed to a high degree (3.8–4 out of 5), while addressing some of the other highest priority institutional challenges, such as funding, facilities, and infrastructure is not an objective of this specific scheme.

Data gathered in the external evaluation allow identification of expected benefits and unexpected benefits to participation in the CDRF scheme from the perspective of fellows and home institutions, as well as potential barriers to stakeholders profiting from these benefits.

In terms of expected benefits, data from the evaluation confirm that fellows perceive an improvement of their clinical research competencies, as defined by the TDR Global Competency Framework for Clinical Research, a result clearly aligned with the objectives and expected outcomes of the one-year training. However, in the context of the ongoing efforts to move health research to LMIC, it is clear that while highly trained clinical research professionals form an important part of making that goal a possible, training researchers will not be sufficient in itself [13]. The factors that contribute to health research concentration in HIC are complex and include multiple institutional, policy, and power dynamics that training at the individual level cannot fully address [14].

The unexpected benefits to participation in the scheme were detected mainly in stakeholder interviews and from information collected during site visits. In this context, fellows and home institutions emphasized the value of knowledge translation competencies and transferable skills developed in the context of the CRDF scheme. The acquisition of these professional skills, in the terminology of the TDR Clinical Research Competency Framework [9], including scientific writing, grant writing, scientific and community communication, and management and leadership skills (scientific meeting management, team leadership, networking, training, and mentoring, study coordination, and participation in scientific and ethical committees) was highly valued by stakeholders. While clinical research competencies are essential, professional transferable skills, including those in scientific leadership, knowledge transfer, and science management, are also necessary for scientists building research careers [15–17].

The main barriers were identified in the return of fellows to their home institutions. Ideally, returning CRDF fellows will find a scientific environment at their home institutions that enables their continued professional growth and development. The lack of this institutional environment can serve as a barrier for individual scientists fully benefitting from their CRDF scheme experience. At the same time, home institutions will only benefit from the scheme if they retain returned fellows and, further, if they have the institutional capacity, facilities, and equipment to manage the translation and dissemination of fellows’ newly acquired competencies to colleagues, both through direct training and the development of new clinical research projects. The availability of local senior researchers with the capacity to not only supervise but more importantly, continuously mentor, could also support the reintegration and professional development of fellows [18]. In the absence of this capacity to benefit from the participation, home institutions may have little interest in engaging actively with the CRDF scheme or developing ownership of it as a key partner.

In this sense, the implementation of the CRDF re-entry grant scheme, a recommendation from the previous external evaluation, in 2014, has served as a useful approach to provide support to both fellows and home institutions, specifically regarding the development and implementation of capacity strengthening activities and vibrant scientific environments.

The analysis of equity within the CRDF scheme, in terms of both gender and the diversity of the participating research institutions, has been addressed to a degree by TDR since last evaluation, but still requires further attention to actively engage the participation of women scientists as well as that of the diverse range of institutions that could enjoy the benefits of this capacity strengthening Scheme.

## Conclusions and recommendations

Providing young researchers with research methodology and technique is not enough if the goal is to achieve well-developed and competitive research systems at country and regional level. Scientific management, leadership, and knowledge translation competencies are increasingly necessary for the professionals who will advocate for science, establish national or international collaborations, and attract funds to build facilities and develop research projects and clinical studies relevant to the public, national policymakers, and other stakeholders; motivate, train and mentor younger colleagues; publish and communicate research results at the international level; and form part of national and international scientific committees, boards, networks, and other decision-making bodies. The development of a robust framework for these competencies, built on the professional skills identified in the TDR Clinical Research Competencies Framework, and a continued focus within the scheme on their development is recommended.

In this same way, while supporting clinical researchers as they develop their careers can be both valuable and effective, this training approach is not sufficient to generate institutional impacts. In the evaluation, home institutions reported benefits to the participation of their staff members as fellows in the scheme, but the evaluators received a generally low response from these institutions and found that, despite improvements made over time, home institution representatives assigned to liaise with the CRDF did not always consistently understand their role within the scheme. High staff turnover and lack of continuity in roles as supervisors and mentors of fellows has an important impact on the institutional ownership of the scheme. Dedicating efforts to communicating with and supporting the home institution scientists who hold these key roles during and after the fellows’ training placements might result in a higher level of engagement and continuous mentoring.

The CRDF re-entry grants to develop research capacity-strengthening activities at institutional level were an important step towards engaging home institutions; however, as mentioned by some fellows, a similar programme of research seed funding would be a welcome addition, beneficial for both individuals and institutions.

At a broader level, given the wide range of factors that may contribute to how likely an institution is to participate in the CRDF scheme, one recommendation for TDR would be to perform a regional-level analysis of research needs as a step toward developing an equity strategy for attracting new home institutions.

Finally, to increase the institutional impact envisioned for the CRDF to the extent that it already realises individual impact, engagement with home institutions should continue to be emphasized with the aim of making them active partners in the scheme jointly with TPO partners. This approach could serve to provide critical support for researchers as they continue to build their careers and, ideally, to maximise the relevance, effectiveness, and impact of the scheme.

## Paper context

To improve the experience and outcomes of capacity strengthening activities, for both individual participants and institutions, it is important to regularly evaluate their implementation and results and to generate recommendations based on these exercises. Publicly sharing the knowledge generated through evaluations promotes transparency and contributes to the collective learning of the global health training community. The TDR CRDF scheme, discussed in this paper, has offered clinical competencies training to mid-career scientists from LMIC since 1999.
